# The western norway mental health interface study: a controlled intervention trial on referral letters between primary care and specialist mental health care

**DOI:** 10.1186/1471-244X-11-177

**Published:** 2011-11-14

**Authors:** Miriam Hartveit, Eva Biringer, Kris Vanhaeht, Kjell Haug, Aslak Aslaksen

**Affiliations:** 1 Research network of Integrated Care in Western Norway, Helse Fonna HF, Haugesund, Norway; 2 Department of Public Health and Primary Care, Faculty of Medicine and Dentistry, University of Bergen, Bergen, Norway; 3 Research section, Division of Mental Health Care, Helse Fonna HF, Haugesund Norway; 4 Center for Health Services and Nursing Research, School of Public Health, Faculty of Medicine, Catholic University Leuven, Leuven, Belgium; 5 European Pathway Association, Belgium; 6 Division of Radiology, Haukeland University Hospital, Bergen, Norway; 7 Faculty of Medicine and Dentistry, University of Bergen, Bergen, Norway

## Abstract

**Background:**

Referral letters are the main communication means between Primary and Specialised Mental Health Care. However, studies of referral letters reveal that they lack important information, and how this lack of information affects the care for patients is unknown. This study aims to explore if and to what degree the quality of referral letters within Mental Health Care for adults can be improved and the potential improvement's impact on defined patient, professional and organisational related outcomes.

**Methods and design:**

A controlled study with pre and post test will be prepared and accomplished to explore the correlation between the content of referral letters and outcomes of the care for the referred patients. The study is performed in accordance with the guideline of the Medical Research Council on development and evaluation of complex interventions. Using a mixed method design, a stepwise model will be conducted: Firstly, process and outcome measures will be developed and tested. Secondly, by these measures, the results from an intervention group of General Practitioners (GPs) who receive a complex quality improvement intervention will be compared with results from a control group who perform "care as usual". Compliance to the introduced guideline will be measured as a mediator.

**Discussion:**

The Western Norway Mental Health Interface Study is among the first trials to evaluate the impact of the quality of referral letters on the organization of care. This study will provide information that will be usable for healthcare managers and clinicians in both Primary and Specialised Care settings.

**Trial Registration:**

ClinicalTrials.gov: NCT01374035

## Background

The prevalence of mental disease is high. Depression is ranked as the leading cause of disability and affects around 120 million people worldwide [1]. As in most countries, Norwegian mental health care is organized using a decentralized model with Primary Health Care often being the first service the patient contacts before being referred to Specialized Mental Health Services. The present decentralisation, sub-specialization and organisation where health professionals, to some degree, independently make decisions regarding the treatment of the patients increases fragmentation in health care [2]. Therefore, the communication and coordination between the various services is essential. It is particularly important in mental health care for three reasons: Firstly, it is composed of multiple providers and services [3], secondly, it consists of interventions that are mutually dependent for achieving a positive outcome for the patient [3] and thirdly, the patients who use mental health care services often have low level of functioning and lack the ability to ensure that they receive the interventions they need [4].

Research shows that the risk of adverse advents is highest during the transition between two links in the process. This is the moment where responsibility for a patient transfers from one service to the next [4,5]. Referral and discharge letters are the most common, and often the only, communication between Primary Care and hospitals [6-8]. However, national and international studies of the quality of this written communication reveal that the quality is poor with regard to the various types of information they cover [7,9-12]. Even though there is no standard for the content of mental health referral letters in Norway, studies imply that there is a potential for improvement also within Mental Health Care [13]. Research has shown that referral letters lack information on assessment of suicidality [13,14], medical and treatment history [15] and planning for integrated care [13].

Quality in Health Services is defined by Øvretveit as "fully meeting the needs of those who need the service most, at the lowest cost to the organisation, within limits and directives set by higher authorities." [16]. It implies that three dimensions are involved: client-quality, professional quality and management quality [16]. When assessing and improving quality, all three dimensions are relevant. "Care Pathways", also known as "Critical Pathways" or "Clinical Pathways", is a complex intervention used to improve the three dimensions in quality of care [17]. Research shows promising results on the effects on patient care and the organization of the care in surgical and medical care when Care Pathways are applied [17-19]. Though there is little research that can demonstrate positive effect of Care Pathways in Mental Health Care [20,21] and in the continuum of care including Primary Care [22,23], the concept is seen as an important contribution toward improving future health care [2,24]. Challenges within research methodology can be argued to be a reason for the limited knowledge on the method's potential and problems [25]. The emphasis on facilitation of communication and coordination in the Care Pathways model implies it has potential to improve the quality of the referral process and letters [26].

A complex intervention is recognized by the high number of interacting components it has. It is made up of a set of components that may interact and cause a synergy effect, which makes it difficult to define the "active ingredients" of the intervention [25,27]. Intervention in the process of coordination and communication between the involved services in a referral process meets the criteria for a complex intervention. The state of the art framework to develop and evaluate complex interventions is described by the Medical Research Council [27,28].

Given the extensive and sole use of referral letters as a link from Primary to Specialised Mental Health Care, it is surprising that their potential for improvement and impact on the service has not been explored to a larger degree. Based on the theoretical and empirical background defined above, there is support for conducting research on these documents' content, the effect they have on the organisation of Specialised Care and the effect of the interventions aimed at improving their content. The increasing use of electronic patient records and electronic transmission of referral and discharge letters is an opportunity for the implementation of research-based interventions that effectively improve and standardise this vital interface [29].

## Methods and design

### Objectives

The main object is to study the function of referral letters as a means to coordinate the care process for adults when referred from Primary Care to Specialised Mental Health Care. The study will explore if and to what degree the quality of these referral letters can be improved and the potential improvement's impact on defined patient, professional and organisational related outcomes.

### Research questions

**Figure 1:**
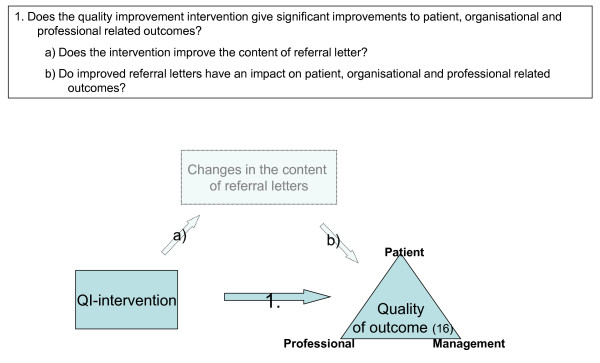
**The main research questions in the study**.

The main research question is to what degree a defined quality improvement intervention geared toward improving the content of referral letters has an impact on patient, professional and organisational related outcomes in the Specialised Mental Health Care. To be able to answer this question we need to ask two underlying questions: Firstly, does a defined quality improvement intervention improve the compliance to the key characteristics of good referral letters? Secondly, what is the correlation between the compliance to the key characteristics of good referral letters and patient, professional and organisational outcomes within the Specialised Mental Health Care? (Figure 1)

There are two premises that are required in order to answer the posed research questions. The first premise is to define the necessary characteristics of a good referral letter to Specialised Mental Health Care and to translate these characteristics into a valid instrument to measure the quality of these letters. The second premise is to define a set of valid outcome measures that are sensitive to the possible impact of the intervention.

#### Design

**Figure 2:**
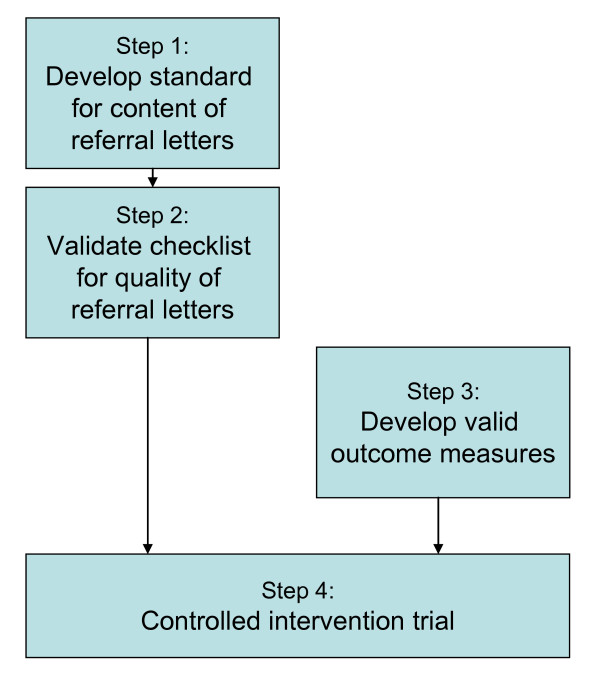
**Illustration of the stepwise progression of the study**.

The study includes the two first phases in Medical Research Council's revised framework for developing and evaluating complex interventions: 1) the "Development" phase and 2) "Feasibility and piloting" phase [28]. Our study consists of four steps performed with a mixed method design that combines qualitative and quantitative approaches in order to answer the research questions. Because of the stepwise progression of the study, each step is planned based on how it will be conducted, but the amount of tests and participants will be decided consecutively based on power analysis and other considerations. Step 1 gives input for development of the characteristics checklist for good referral letters. In step 2, the checklist's validity as an instrument for assessing the quality of referral letters will be tested. During step 3, the set of outcome measures will be developed and tested to strengthen the causal chain [28]. Step 4 consists of a quasi-experimental study with a pre-post test design using an intervention and a control group (Figure 2).

Step 1: A qualitative study with the aim of detecting the characteristics of good referral letters and outcomes that could be affected by improved referral letters will be performed. Interview by nominal group technique [30] in groups with representatives of the patient, professional (mental health nurses and GPs in Primary Health Care and psychiatrists and psychologist in Specialised Mental Health Care) and management perspectives will be conducted. The groups will be asked questions regarding the two premises: A) Information referral letters should contain to give the Specialised Mental Health Care the necessary information to correctly and sufficiently prioritize, plan treatment and follow-up the patients and B) The possible impact improved referral letters could have on Specialized Mental Health Care.

Step 2: The main aim in step 2 is to use the results from step 1 and premise A together with the results from a literature search to develop a valid tool to assess the quality of referral letters. A Delphi-technique [30] will be used to rank the characteristics defined in step 1 and defined in the literature on content of referral letters. Both the participants in all the interview groups and specialists from Specialised Mental Health Care and general practitioners (GPs) will be included in this study phase. The alpha version of the tool will be tested on psychometric properties in terms of their interrater reliability, test-retest reliability and correlation between checklist score and receivers' assessment of the referral letter's usefulness. The reliability tests are to be performed on referral letters drawn from a retrospective sample of patient records from Helse Fonna local health trust, Division for Mental Health Care. The number of documents examined by the checklists will be determined by a power analysis after the checklist is developed and pilot testing (N = 10) is completed.

Step 3: The main aim in step 3 is to develop a set of valid outcome measures that are sensitive to changes in Specialised Mental Health Care following improved referral letters. The development of measures will be based on a triangulation of methods [31]. A set of possible measures will be developed based on the interview groups' suggested measures and a literature review on process and outcome measures relevant for Specialised Mental Health Care. These measures will be tested one by one on their correlation with the quality of the referral letter. A number of referral letters will be drawn from the Electronic Patient Record System, depersonalized and scored on the developed referral letter checklist. For each referral letter, data on the suggested outcome measures will also be collected. The correlation between quality of referral letters and outcome measures will then be tested. The outcome measures that are found to have the strongest theoretical and empirical support for their correlation with the quality of referral letters will be used during the intervention study in step 4.

Step 4: The aim of step 4 is to study, firstly, if and to what degree a Care Pathway-inspired intervention for GPs improves the compliance to the guideline developed in step 1 and 2 and, secondly, what the impact is of the intervention and improved referral letters on patient, organisational and professional related outcomes. A controlled quasi-experimental design with pre and post test will be conducted.

#### Setting and Sample

**Figure 3:**
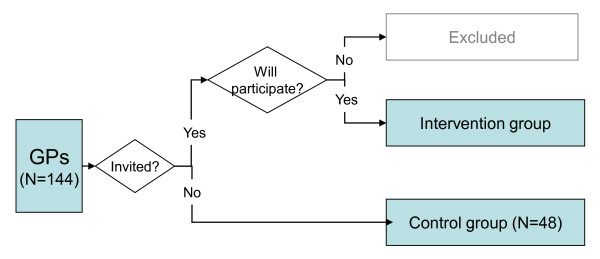
**Selection of the sample**.

The study will be conducted within the region of Helse Fonna Local Health Authority on the Western coast of Norway. This health authority is responsible for the Specialised Health Care of 18 municipalities and has a total population of 165,000. Four public local mental hospitals and two public specialised mental health hospitals constitute most of the Specialised Mental Health Care for the population. Mental Health Services in Helse Fonna receives approximately 300 referral letters per month. There are 144 GPs within the region. To reduce the risk for contamination of the data, GPs within a health centre or office will be seen as a cluster in the inclusion process. All GP offices will be divided into two groups: one will be invited to participate and the second will serve as a control group. GPs in an invited centre who volunteer to participate constitute the intervention group. Data from GPs who do not choose to participate, but are working with participants in the intervention group, will be excluded (Figure 3).

#### Units of analysis

To answer the research question regarding the impact of the intervention on the quality of the referral letters, the units of analysis will be each GP. When studying the intervention's impact on the outcome, each referral letter will form the unit of analysis. Only referral letters for elective examination or treatment will be included.

#### Description of the intervention

The intervention includes several components to enhance the mutual understanding of the referral process by making the different activities, roles and goals explicit [32]. It meets the criteria for a complex intervention and constitutes a set of intervention elements adapted into the context of the organization [27,28,33] to facilitate the phases in the process of change defined by Grol and Wensing [30]. The intervention includes development of process and outcome measures defined in step 1-3. Firstly, GPs in the intervention group, in cooperation with representatives from Primary Mental Health Care, Specialist Mental Health Care, patient representatives and managers, will be involved in defining the key characteristics of a high quality referral letter to Specialised Mental Health Care Secondly, the participants in the intervention group will be presented the characteristics of a good referral letter, data on compliance to these characteristics in existing referral letters and the outcomes following the referral letters. Thirdly, they will participate in an individual interview focusing on ability and motivation for change. This is mainly seen as a part of the data collection, but can also serve as an active ingredient. And finally, they will receive consecutive feedback on their performance when they send a referral letter, both on the compliance to the guidelines and the outcome for the patient.

#### Description of the measures

Following the MRCs guidelines for complex interventions, this study will make use of both qualitative and quantitative data to answer the research questions. Within step 1 and 2, a validated checklist to assess the quality of referral letters will be developed as a process measure. Outcome measures will be developed in step 3. In addition, data on the process of implementing a change and the context will be collected both by questionnaire and individual interviews. The data collection consists of the following:

##### Structure measures

Before the intervention:

• Questionnaire to the GPs in the intervention group on background variables (age, experience as GP, approximate number of patients with moderate or severe mental health problems, etc.).

• Individual interview with the GPs in the intervention group about their experience with structured quality improvement efforts and referrals to Specialised Mental Health Care, their motivation for changing and their plan to implement the new guideline.

After the intervention:

• Individual interview with the GPs in the intervention group about their experience with the intervention and motivators for continuous improvement.

##### Process measures

• Quality of referral letters measured by the validated checklist from step 1 and 2.

##### Outcome measurement

• Quality of care measured by the indicators from step 3.

• Length of stay. Measurement is from date of admission or onset of out-patient treatment until date of discharge (documented in Electronic Patient Record as end of treatment period), assessed up to six months after admission.

• Response time for referral letters in Specialised Mental Health Care. Referral letters will be followed for the duration of the assessment and prioritization process until response letter is sent, an expected average of 10 days.

• Usefulness of the information in the referral letters. Based on the specialist's assessment, the usefulness and accuracy of the information given in the referral letters will be scored after the first consultation with the patient.

**Figure 4:**
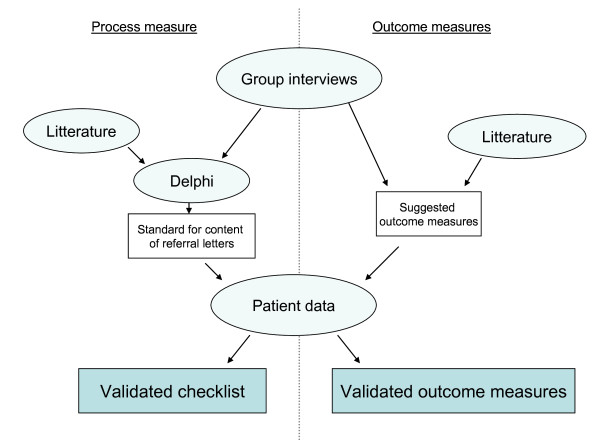
**The development of the process and outcome measures**.

The data collection will mainly be prospective. However, since the development of the process measures a part of the intervention, the pre test of compliance to the characteristics of high quality referral letters will be retrospective using referral letters from the Patient Record System (Figure 4).

### Registration and Ethical approval

Step 1 and 2 are approved by the Norwegian Social Science Data Service (register number 24340) and the National Committee for Medical and Health Research Ethics. The application for approval for steps 3 and 4 depends on steps 1 and 2, and will be applied for after step 2 is complete. The study is registered at ClinicalTrials.gov registration number NCT01374035.

Participation will be based on written informed consent. All data from the Patient Record System, including referral letters, will be depersonalized by health professionals who already have access to the information before it is delivered to the researchers.

## Discussion

Cooperation and coordination is necessary for ensuring that the patients in Mental Health Care receive a sufficient continuum of care when referred from Primary to Specialist Health Care. However, our knowledge about the degree to which the main means for this - the referral letter - affects the specialised care is limited. Because of this, we do not know whether we should place emphasis on improving the referral letters. The main object of this study is to investigate the impact of an intervention aimed at improving the quality of referral letters on patient, professional and organisational related outcomes. The intervention is a complex intervention. The implementation of such a complex intervention, according to the MRCs definition, requires careful planning and development of measurements that can detect important causal chains. The study employs a stepwise development of valid process and outcome measures.

Triangulation is recommended to enhance the validity of the findings to compensate for the methods' various weaknesses [31]. We combine three research methods by making use of data from group interviews, examining existing literature and testing existing data from patient records to develop process and outcome measures. From these procedures, we will be able to perform a controlled intervention study in Mental Health Care for adults. Since the strong focus on referral letters as important means for care coordination can be argued to be based mainly on expectations and beliefs rather than evidence, this study will contribute important information about referral letters even if we find no correlation between improved referral letters and quality of care.

There are several possible obstacles to effective cooperation between Primary Care Services and Specialist Mental Health Services [34]. There may not be agreement between the services about which information is essential and correct in referral letters. This study will, therefore, use a guideline for content of referral letters that will be collaboratively developed by representatives from both Primary and Specialist Health Care. We will also test the correlation between the quality of a referral letter defined by the guideline to the quality defined by the specialist's assessment after the first consultation with the patient.

In this study we will be able to compare the intervention group's pre results with the post results as well as compare the results of the intervention group with the control group. The intervention group consists of GPs who are willing to participate and the control group is expected to be a natural sample of GPs. We do not expect them as group to differ in the way they write referral letters before the intervention. But we expect that the intervention group could be more motivated for change than the control group as defined in Prochaska's model for stages of readiness to change [30]. Since we compare content of referral letters, not willingness to change, we argue that the groups are comparable. However, we emphasise that the intervention is tested on a group expected to be at the "contemplation" or a later phase in Prochaska's model. The effect of the intervention can only be generalized to groups that are at the same level of readiness, not to the general population.

A lot of effort is put into improving the coordination between health services. The referral letters are seen as an important means for this coordination, and they have been found to lack important information. However, we do not know if and how the quality of these letters affects the outcome for the patients and the services. This study will explore the correlation between the quality of referral letters and outcome measures based on the state-of-the-art framework of the Medical Research Council. To our knowledge, this is the first trial about the impact of referral letters on Specialised Mental Health Care and is an important aspect of building knowledge about a complex process of coordination and possible improvement potential within Health Care.

## List of abbreviations

MRC: Medical Research Council; QI: Quality improvement; GPs: General Practitioners (medical doctors in Primary Health Care)

## Declaration of competing interests

The authors declare that they have no competing interests.

## Authors' contributions

The paper was written by MH, with supervision from EB, KV, KH and AA. All authors have contributed to the development of the protocol. KV made particularly helpful contributions in methodology and publishing. In addition to participating in the development of the overall plan, EB leads the research network that funds this study. All authors have read and approved the final manuscript.

## Pre-publication history

The pre-publication history for this paper can be accessed here:

http://www.biomedcentral.com/1471-244X/11/177/prepub
